# Genome Sequence of *Pseudomonas mandelii* PD30

**DOI:** 10.1128/genomeA.00713-14

**Published:** 2014-07-17

**Authors:** Philip A. Formusa, Tom Hsiang, Marc B. Habash, Hung Lee, Jack T. Trevors

**Affiliations:** School of Environmental Sciences, University of Guelph, Guelph, Ontario, Canada

## Abstract

The genome sequence of *Pseudomonas mandelii* PD30 is reported in this announcement. The genes for the reduction of nitrate to dinitrogen were identified in the genome assembly and subsequently used in gene expression research.

## GENOME ANNOUNCEMENT

*Pseudomonas mandelii* was first isolated from mineral water in France and was identified as belonging to the *P. fluorescens* group (1). *P. mandelii* was also identified in mineral water samples from South Korea, and in soil samples from China, the United States, and Canada (2–4).

*P. mandelii* PD30 was isolated from agricultural soil samples collected at potato fields in New Brunswick, Canada, where it was identified as a dominant culturable denitrifying bacterium (2). In pure cultures of *P. mandelii* PD30, anaerobic nitrite and nitric oxide reductases were observed to be optimally expressed at 30° C and pH 7.0 (5). *P. mandelii* PD30 was used to investigate the influence of carbon and nitrate amendments on the expression of denitrification genes encoding the catalytic reductases (6).

Genomic DNA was isolated from overnight cultures grown aerobically in 5 mL of Tryptic soy broth at 28°C and shaken at 180 rpm. Following overnight growth, genomic DNA was extracted using the DNeasy Blood and Tissue kit (Qiagen Sciences, Germantown, MD). Two micrograms of DNA were sequenced using an Illumina HiSeq 2000 platform and 100-bp paired-end reads in one-tenth of a lane. A total of 26,662,271 reads were generated, giving a genome sequencing coverage above 7,000×. Genome assembly was done using the Linux-based SOAP *de novo* (version 1.05) and GapCloser (version 1.1.2). The assembly generated 1923 contigs, 110 of which were larger than 200 bp. The draft genome with contigs greater than 200 bp was 6.7 Mb in size, with an average G+C content of 59.0%. The NCBI prokaryotic genome pipeline (GeneMark S+) was used to predict a total of 6,129 protein-encoding genes, 61 tRNA genes, and 9 rRNA genes.

Denitrification genes for complete reduction of nitrate to dinitrogen were identified in the genome assembly. In addition to the membrane-bound nitrate reductase gene cluster (*nar*), a periplasmic (*nap*) and assimilatory (*nas*) gene clusters were identified. Similar to *P. fluorescens* F113 (7), a second nitric oxide (*nor*) and nitrous oxide (*nos*) reductase gene clusters were identified.

### Nucleotide sequence accession number.

The draft genome sequence of *P. mandelii* PD30 has been deposited in NCBI under the accession number AZQQ00000000.
